# Supplementary material for: Cellular and synaptic organization of the Octopus vertical lobe
Source: bioRxiv. 2025 Jan 29:2025.01.29.635406. Preprint. [Version 1] doi: 10.1101/2025.01.29.635406 (PMC11838284; doi:10.1101/2025.01.29.635406)
Supplement: 1 [file NIHPP2025.01.29.635406V1-supplement-1.pdf]

bioRxiv preprint doi: https://doi.org/10.1101/2025.01.29.635406; this version posted January 29, 2025. The copyright holder for this preprint (which was not certified by peer review) is the author/funder, who has granted bioRxiv a license to display the preprint in perpetuity. It is made available under aCC-BY 4.0 International license.

``

Supplementary figure

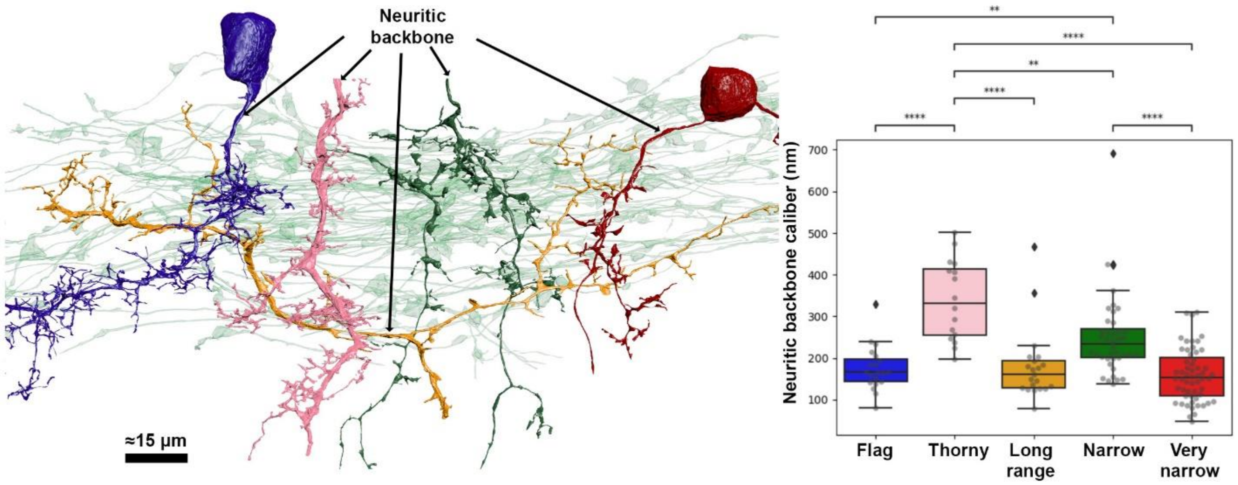

Figure S1: **The 5 morphological subtypes of CAMs display different backbone diameters, suggesting variations in their electrical properties.** A. Reconstructions of CAMs, color-coded by subtype, are overlaid on the SFL tract (green). CAMs exhibit a wide array of morphological subtypes. These bifurcate into two primary classes: horizontal cells, which include the flag (Fl, in blue) and putative long-range (Lr, in orange) cells, and vertical cells, which comprise the narrow dendritic (Nw, in red), very narrow dendritic (Vw, in green), and thorny (Th, in pink) cells. The neuritic backbone significantly varies among subtypes (Kruskal-Wallis test, $p<0.001$). A permutation test with Bonferroni correction indicated pairwise differences. Levels of statistical significance are denoted by asterisks: *** $p<0.0001$, ** $p<0.001$, * $p<0.005$.
